# A rare case of sigmoid colon cancer in which the lower limbs received collateral blood flow from the inferior mesenteric artery owing to peripheral artery disease

**DOI:** 10.1186/s40792-021-01274-9

**Published:** 2021-08-21

**Authors:** Kyoichi Kihara, Hiromu Horie, Kozo Miyatani, Masayuki Endo, Tomoyuki Matsunaga, Manabu Yamamoto, Shinsaku Yata, Naruo Tokuyasu, Teruhisa Sakamoto, Yoshiyuki Fujiwara

**Affiliations:** 1 Division of Gastrointestinal and Pediatric Surgery, Department of Surgery, School of Medicine, Faculty of Medicine, Tottori University, 36-1 Nishimachi, Yonago, Tottori 683-8504 Japan; 2 Division of Cardiovascular Surgery, Department of Surgery, School of Medicine, Faculty of Medicine, Tottori University, 36-1 Nishimachi, Yonago, Tottori 683-8504 Japan; 3 Division of Radiology, Department of Multidisciplinary Internal Medicine, School of Medicine, Faculty of Medicine, Tottori University, 36-1 Nishimachi, Yonago, Tottori 683-8504 Japan

**Keywords:** Peripheral artery disease, Arteriosclerosis obliterans, Colorectal cancer, Inferior mesenteric artery, Collateral artery, Angiography, Limb ischemia, Lower extremity, Indocyanine green fluorescence, Multidisciplinary

## Abstract

**Background:**

Colorectal cancer and peripheral artery disease are common conditions in older adults and may coexist in this population. Lymph node dissection along the inferior mesenteric artery is a vital procedure in cases of left-sided colorectal cancer. However, the inferior mesenteric artery may show a collateral blood pathway in rare cases of peripheral artery disease. We report a case of advanced sigmoid colon cancer in which the lower limbs received inferior mesenteric artery flow owing to asymptomatic peripheral artery disease. The possibility of catastrophic lower-limb ischemia because of complete mesenteric excision with ligation of the inferior mesenteric artery was a matter of concern in this case.

**Case presentation:**

A 73-year-old man with asymptomatic peripheral artery disease was diagnosed with stage IIIB advanced sigmoid colon cancer. Angiography using a balloon-occlusion catheter revealed that his lower limbs received prominent inferior mesenteric artery blood flow through a collateral pathway. Therefore, interventional radiologists and cardiovascular surgeons evaluated the indications for endovascular stents or bypass grafts. The patient also had dilated cardiomyopathy, so the cardiovascular physicians evaluated his tolerance in the worst-case scenario of a colorectal anastomotic leak. The patient underwent axillofemoral artery bypass and two-stage laparoscopic sigmoid colectomy without anastomosis. The postoperative course was uneventful, and he resumed his job within a month after the resection.

**Conclusions:**

Although collateral flow from the inferior mesenteric artery is rare in patients with peripheral artery disease, a few case reports have described fatal lower-limb ischemia following anterior resection. The perioperative multidisciplinary evaluation enabled us to understand the patient’s condition and risks, and allowed successful cancer treatment without ischemia of the lower limbs.

## Background

The increasing population of older adults worldwide has resulted in a greater incidence of colorectal cancer (CRC) as well as an increase in the number of people living with atherosclerosis [1]. Peripheral artery disease (PAD), also known as arteriosclerosis obliterans, is caused by plaque buildup in the abdominal aorta and iliac arteries, which leads to stenosis of the vessels that deliver blood from the heart to the legs. The inferior mesenteric artery (IMA) is a rare collateral feeding artery in PAD [2, 3]. Lymph node dissection along the IMA is a vital step in cases of left-sided CRC. We report a patient with advanced sigmoid colon cancer whose lower limbs received IMA blood flow owing to asymptomatic PAD. The possibility of catastrophic ischemia of his lower limbs because of complete mesenteric excision (CME) with ligation of the IMA was a major concern in this case, and a multidisciplinary approach played an important role in both the success of cancer treatment and the avoidance of catastrophic ischemia of the lower limbs.

## Case presentation

**Fig. 1:**
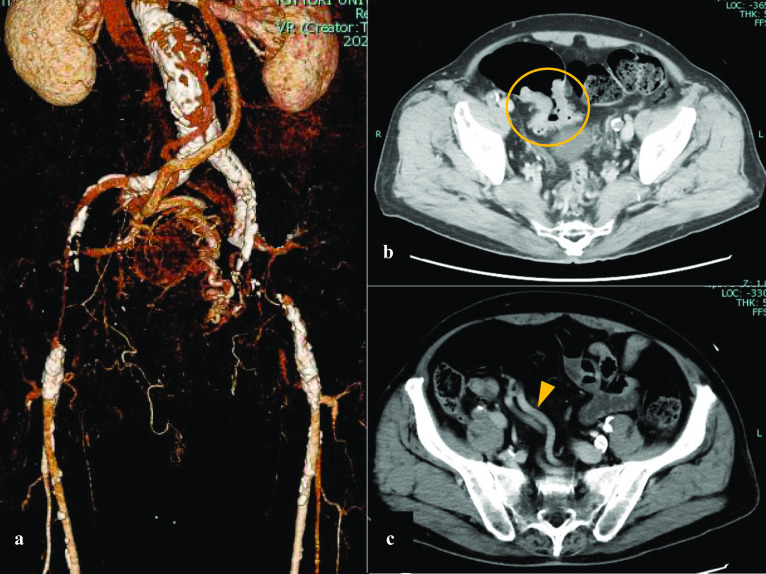
Computed tomography images from periodic surveillance for abdominal aortic aneurysm and asymptomatic peripheral artery disease. **a** Severe calcification was observed between the abdominal aorta and both femoral arteries. Both external iliac arteries were disrupted. **b** Well-demarcated wall thickening was found in the sigmoid colon. **c** The inferior mesenteric artery was well developed and measured more than 5 mm in diameter. A dilated inferior mesenteric vein was winding along the inferior mesenteric artery

**Fig. 2:**
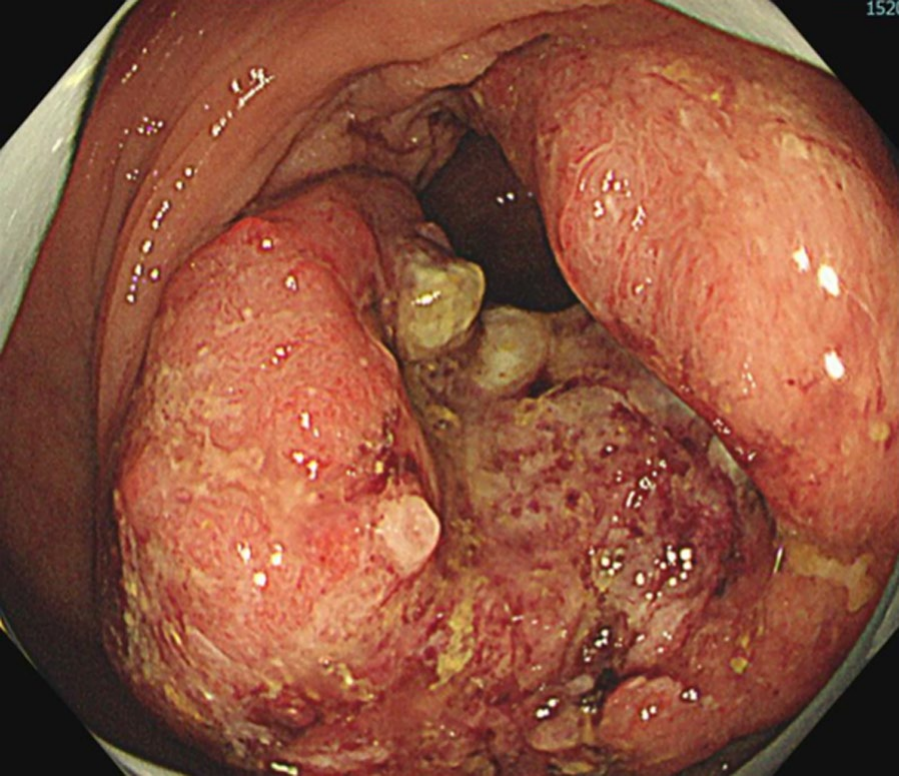
Colonoscopic image of advanced sigmoid colon cancer. The tumor was categorized as type 2 and occupied four-fifths of the circumference. The scope did not pass through the cancer canal. Well-differentiated adenocarcinoma was evident from the biopsy specimen

**Fig. 3:**
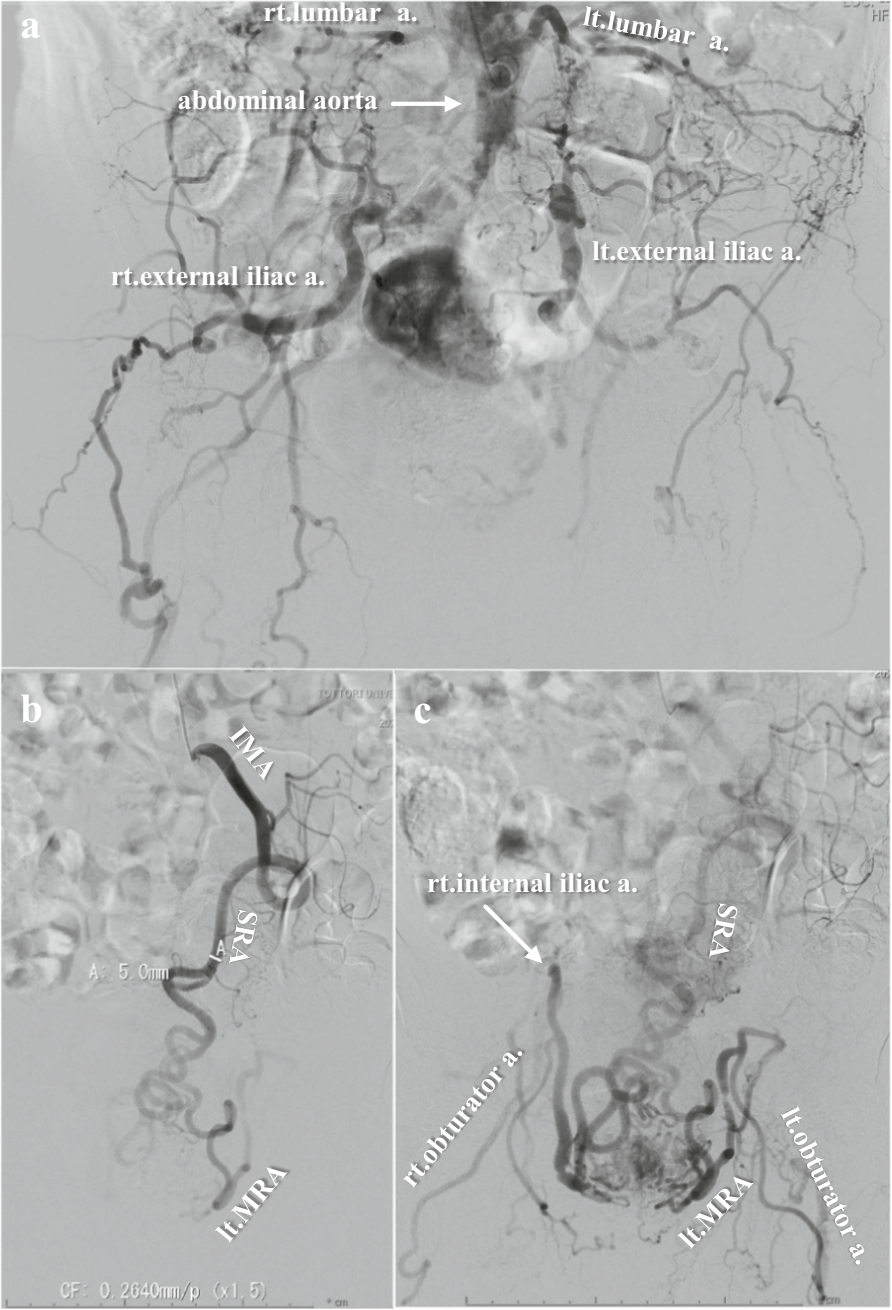
Images from angiographic evaluation by interventional radiology. **a** Abdominal aortography with inferior mesenteric artery (IMA) balloon occlusion showed developed collateral lumber arteries feeding the lower extremities. **b** The early phase of IMA selective angiography showed a prominent IMA flow. **c** In IMA selective angiography, retrograde blood flow of the internal iliac arteries was sustained by IMA blood flow through the collateral mid-rectal arteries

**Fig. 4:**
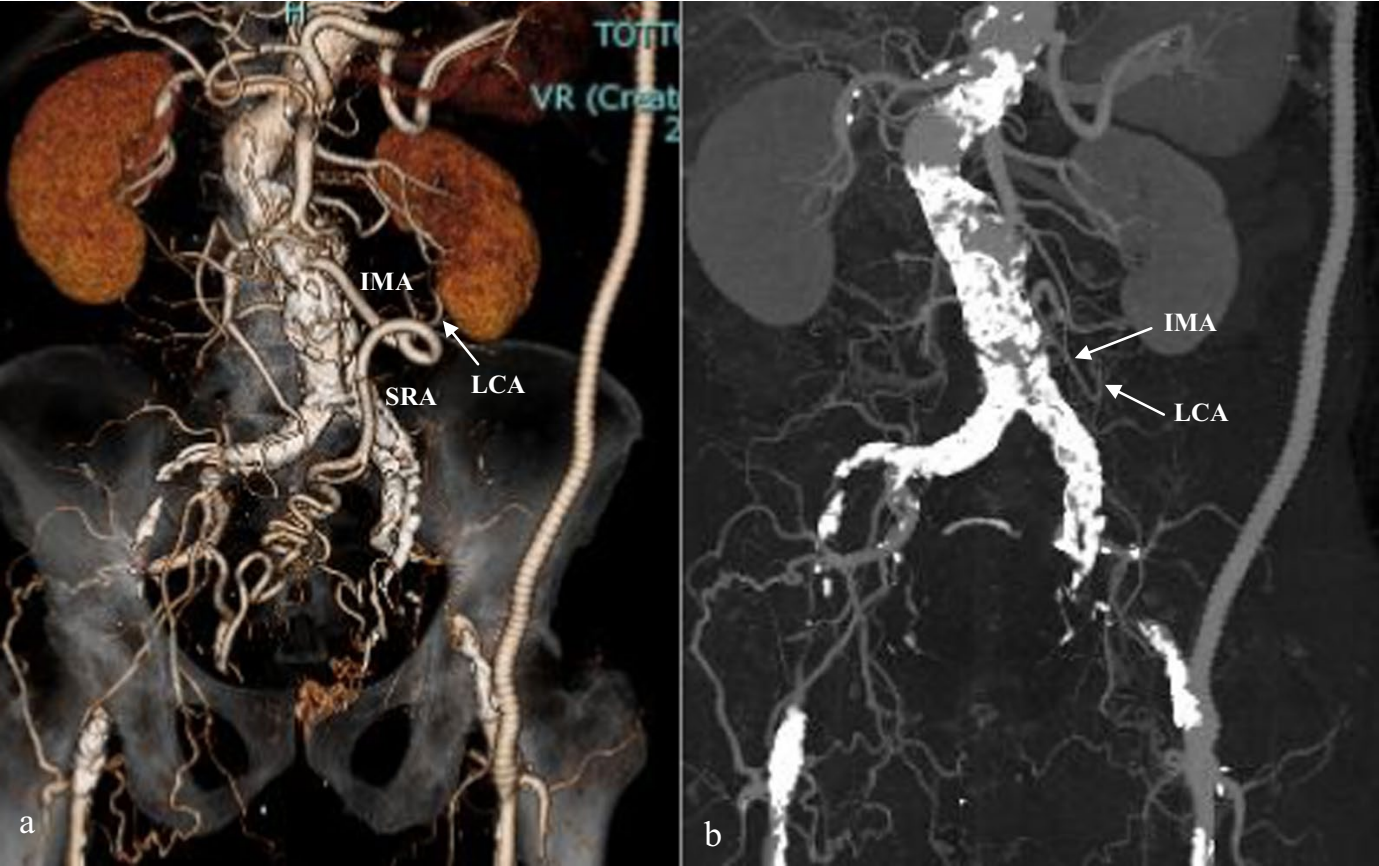
Computed tomography angiography taken after the surgeries. **a** After the axillofemoral bypass. Excellent blood flow to the left extremity through the bypass was proven. **b** After the laparoscopic sigmoid colectomy with inferior mesenteric artery preservation, composed of preservation of the inferior mesenteric artery to left colic artery and ligation of the superior rectal artery

**Fig. 5:**
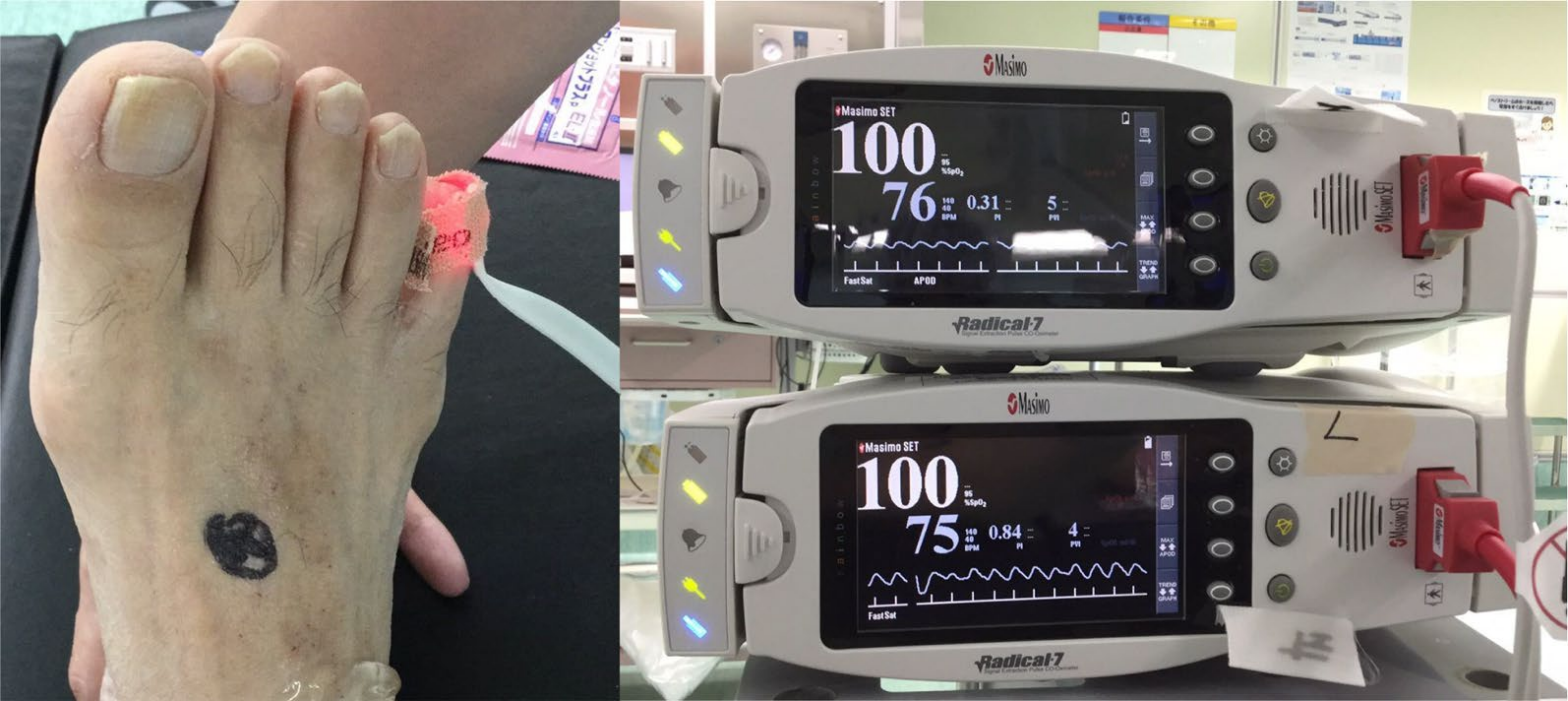
To visualize sufficient blood flow to the lower extremities, oxygen saturation was monitored in both feet during laparoscopic sigmoid colectomy, which required extended lithotomy in the Trendelenburg position

A 73-year-old man underwent periodic surveillance for abdominal aortic aneurysm (AAA) and asymptomatic PAD at the Division of Cardiovascular Surgery in our hospital. The patient was a skilled, active dentist. His resting ankle–brachial index (ABI) was 0.55 and 0.52 for the right and left sides, respectively. Computed tomography (CT) indicated an AAA of 38 mm, PAD, the possibility of rectal arteriovenous malformation, and the increased wall thickness of the sigmoid colon with regional lymph node swelling (Fig. 1). The IMA was well developed and measured more than 5 mm in diameter on CT angiography. Colonoscopy revealed advanced sigmoid colon cancer (Fig. 2), and our initial diagnosis was cT4aN1bM0, cStage IIIB sigmoid colon cancer according to the 8th edition of the Union for Cancer Control TNM classification. The patient also had dilated cardiomyopathy. Echocardiograms revealed a left ventricular ejection fraction of 35–40% with regular administration of β-blockers, angiotensin-converting-enzyme inhibitors, and diuretics. Two major concerns were related to successful cancer treatment. First, ligation of the IMA or superior rectal artery (SRA), which is a vital procedure in CME for advanced sigmoid colon cancer, could result in insufficient blood flow and ischemia of the lower limbs. Second, the procedure could result in insufficient blood supply to the remaining rectum and increase the risk of leakage in the remaining rectosigmoid anastomosis. In the treatment of symptomatic PAD, CT angiography is a potentially less invasive and adequate technique to plan for the provision of additional blood supply during surgery. However, evaluation of blood distribution after shutting off the IMA blood flow was not sufficient, and further angiographic work-up using a balloon-occlusion catheter by interventional radiologists revealed that his lower limbs were receiving collateral blood flow from the internal iliac branches, with the flow being worse in the left limb. The flows of the internal iliac arteries were sustained by the prominent IMA blood flow through the collateral mid-rectal arteries and collateral lumbar arteries (Fig. 3). In a multidisciplinary conference with interventional radiologists and cardiovascular surgeons, a left axillofemoral bypass graft was preferred to avoid ischemia of the lower limbs. Because of the low patency rate of an axillofemoral bypass graft [4], a “Y-shaped” connection to the bilateral femoral artery was not proposed to preserve the right femoral artery in case of graft occlusion. Additionally, the possibility of conversion from laparoscopic surgery to laparotomy requiring a median hypogastric incision traversing the Y-shaped bypass could not be ruled out. The patient underwent surgical angioplasty of the left common to the superficial femoral artery and axillofemoral artery bypass (Fig. 4a). Another multidisciplinary conference was held among cardiovascular physicians, anesthetists, perioperative nurses, and gastrointestinal surgeons. Retrograde blood flow through the obturator artery to the remaining rectum can be expected after IMA ligation. However, taking the patient’s comorbidities into consideration, the cardiovascular physicians warned that his heart would not overcome the pan-peritonitis if an anastomotic leak occurred. Although indocyanine green fluorescence imaging (ICG-FI) was used intraoperatively to confirm blood supply to the remaining rectum, we proposed Hartmann’s procedure and obtained the patient’s consent. A month after the bypass, the patient underwent laparoscopic sigmoid colectomy and D3 lymph node dissection with IMA preservation, composed of preservation of the IMA to the left colic artery and ligation of the SRA (Fig. 4b). Anesthetists and perioperative nurses proposed oxygen saturation monitoring of both feet during laparoscopic surgery, which required extended lithotomy in the Trendelenburg position (Fig. 5). The mobilization of the rectum was limited down to the peritoneal reflection so as not to damage the middle rectal artery (MRA), which provided collateral blood flow. The MRA fed the patient’s lower limbs until ligation of the SRA and provided retrograde flow to the remaining rectum after the sigmoid colectomy. Although ICG-FI revealed good perfusion of the remaining rectum, anastomosis was omitted as planned. The cardiovascular surgeon evaluated the blood flow at both feet by Doppler echo immediately after surgery in the operating room and confirmed sufficient blood flow to the lower limbs. The patient’s postoperative course was uneventful, and he was discharged 10 days postoperatively. Pathological examination of the specimen showed a pT3N0M0, pStage IIA tumor. The patient resumed work a month after the resection and was followed up for a year with no evidence of tumor recurrence.

## Discussion

We report a successful multidisciplinary approach for a case of advanced sigmoid colon cancer in which the lower limbs received collateral blood flow from the IMA owing to asymptomatic PAD. The classic symptom of PAD is intermittent claudication, defined as pain and cramping in the legs on exertion that is relieved by rest. Diagnosis in symptomatic patients is usually made based on the resting ABI, with ABI values less than 0.90 considered abnormal. However, the prevalence of PAD is unknown because many patients are asymptomatic owing to the development of collateral blood flow [5]. The collateral pathway from the IMA is rarer than those from the lumbar, intercostal, deep circumflex iliac, internal thoracic, and inferior epigastric arteries [2]. Only a few reports have described a collateral pathway from the IMA, and all of them have reported fatal lower-limb ischemia following anterior resection in patients with either asymptomatic or symptomatic PAD [6–8]. Several mechanisms have been considered to explain these findings, including undue pressure on the popliteal artery, acute thrombosis, low cardiac output during operation, and ligation of the IMA as the most relevant factor. However, without angiographic data, the exact sequence of events remains speculative. CT angiography may be valid in surgical candidates with symptomatic PAD [9]. However, the major points in our case were the amount of blood flow borne by the IMA to the limbs and the potential influence of IMA ligation on the possibility of lower-limb ischemia. Angiography using a balloon-occlusion catheter could answer these questions. Angiographic data provide adequately determinative material for cardiovascular surgeons to confirm the use of a bypass graft before sigmoid colectomy.

Some case reports of rectal resection with PAD have advocated that anastomosis should be avoided because of its potentially high risk of leakage due to poor blood microcirculation [10]. On the other hand, ICG-FI during surgical procedures is a well-recognized method for evaluating blood perfusion of the remaining gut and may contribute to reduction of the anastomotic leak [11]. Thus far, the interpretation of ICG-FI data is still more subjective rather than objective. We already had the patient’s consent to construct a sigmoid colon stoma on the grounds that his cardiac function was insufficient in the worst-case scenario of an anastomotic leak. IMA was preserved not for anastomosis but for intestinal peristalsis of the remaining left-sided colon and for avoidance of stoma-necrosis, which is especially relevant in patients with severe atherosclerosis [12].

Surgical resection has been the primary modality in CRC treatment. Less invasive procedures, such as laparoscopic surgery and robot-assisted surgery, have been invented in the last decades, and more elderly patients can now undergo surgery. Perioperative comprehensive evaluation and multidisciplinary approaches are becoming increasingly important for better outcomes. In patients with PAD, angiographic evaluation is important and must be performed before left-sided colectomy or rectal resection to avoid fatal lower-limb ischemia, even in patients with asymptomatic PAD.

## Conclusions

We encountered a case of advanced sigmoid colon cancer in which the lower limbs received IMA blood flow. A perioperative multidisciplinary approach proved very important for a better understanding of the patient’s condition and risks and allowed successful cancer treatment without ischemia of the lower limbs.

## Data Availability

The data used in this report are available from the corresponding author on request.

## Abbreviations

**AAA**: Abdominal aortic aneurysm
**ABI**: Ankle–brachial index
**CME**: Complete mesenteric excision
**CRC**: Colorectal cancer
**CT**: Computed tomography
**ICG-FI**: Indocyanine green fluorescence imaging
**IMA**: Inferior mesenteric artery
**MRA**: Middle rectal artery
**PAD**: Peripheral artery disease
**SRA**: Superior rectal artery
